# Does sex influence the diagnostic evaluation of autism spectrum disorder in adults?

**DOI:** 10.1177/1362361315611381

**Published:** 2016-01-22

**Authors:** C Ellie Wilson, Clodagh M Murphy, Grainne McAlonan, Dene M Robertson, Debbie Spain, Hannah Hayward, Emma Woodhouse, P Quinton Deeley, Nicola Gillan, J Chris Ohlsen, Janneke Zinkstok, Vladimira Stoencheva, Jessica Faulkner, Hatice Yildiran, Vaughan Bell, Neil Hammond, Michael C Craig, Declan GM Murphy

**Affiliations:** 1 King’s College London, UK; 2 South London and Maudsley NHS Foundation Trust, UK; 3 University of Seville, Spain; 4 Bethlem Royal Hospital, UK; 5 Bristol Autism Spectrum Service, UK; 6 University of Bristol, UK; 7 University College London, UK

**Keywords:** autism spectrum disorder, diagnosis, females, males, sex differences

## Abstract

It is unknown whether sex influences the diagnostic evaluation of autism spectrum disorder, or whether male and female adults within the spectrum have different symptom profiles. This study reports sex differences in clinical outcomes for 1244 adults (935 males and 309 females) referred for autism spectrum disorder assessment. Significantly, more males (72%) than females (66%) were diagnosed with an autism spectrum disorder of any subtype (x^2^ = 4.09; *p* = 0.04). In high-functioning autism spectrum disorder adults (IQ > 70; N = 827), there were no significant sex differences in severity of socio-communicative domain symptoms. Males had significantly more repetitive behaviours/restricted interests than females (*p* = 0.001, d = 0.3). A multivariate analysis of variance indicated a significant interaction between autism spectrum disorder subtype (full-autism spectrum disorder/partial-autism spectrum disorder) and sex: in full-autism spectrum disorder, males had more severe socio-communicative symptoms than females; for partial-autism spectrum disorder, the reverse was true. There were no sex differences in prevalence of co-morbid psychopathologies. Sex influenced diagnostic evaluation in a clinical sample of adults with suspected autism spectrum disorder. The sexes may present with different manifestations of the autism spectrum disorder phenotype and differences vary by diagnostic subtype. Understanding and awareness of adult female repetitive behaviours/restricted interests warrant attention and sex-specific diagnostic assessment tools may need to be considered.

## Introduction

Autism spectrum disorder (ASD) is a neurodevelopmental condition diagnosed when there is evidence from early childhood of impairments in social functioning and communication co-occurring with repetitive behaviours and restricted interests (International Classification of Diseases–10th Revision (ICD-10R); World Health Organization (WHO, 1993); *Diagnostic and Statistical Manual of Mental Disorders*, 5th ed. (DSM-5); American Psychiatric Association (APA), 2015). ASD is a common condition with recent epidemiological studies in the United Kingdom estimating prevalence at between 1% (Brugha et al., 2012) and 1.7% (Russell et al., 2014). Males are diagnosed approximately four times more often than females in childhood (Fombonne, 2005, 2009) although this ratio varies with IQ and is reportedly as low as 2:1 when ASD is co-morbid with intellectual disability, and as high as 6–8:1 in high-functioning populations (Fombonne, 2005). The reason for the gender discrepancy is unclear. It has been proposed that females require a greater assault at the genetic level in order to develop ASD (Jacquemont et al., 2014; Levy and Perry, 2011), and that ASD in females may be less frequently diagnosed because females tend to be better at compensating for their difficulties (Attwood, 2007; Lai et al., 2011). Additionally, males and females may present with different ASD phenotypes (Howe et al., 2015; Mandy et al., 2012; Van Wijngaarden-Cremers et al., 2014); this may affect diagnostic rates since the female profile is less well understood and hence less easily detected (Kirkovski et al., 2013). It is important to establish how sex influences the presentation of ASD because this has implications for understanding the biology of ASD in both sexes, and has implications for service design and clinical care. Therefore, we report, to the best of our knowledge, the first large-scale comparison of symptom profiles in men and women who were referred for an assessment of ASD for the first time in adulthood.

Sex differences in children with ASD have been reported in several previous studies. The largest study to date included 304 girls and 2114 boys aged 4–18 years with a diagnosis of ASD (Frazier et al., 2014). They reported that girls showed more social and communication symptoms on the Autism Diagnostic Observation Schedule–Generic (ADOS-G; Lord et al., 2000) and had fewer repetitive behaviour symptoms on the Autism Diagnostic Interview–Revised (ADI-R; Lord et al., 1994). Most participants in the sample had an IQ below 80, but the ASD girls had lower average IQ in both verbal and performance domains than the ASD boys; lower IQ scores in females was advanced as an explanation for greater social impairment but did not mediate fewer symptoms in repetitive interests/restricted behaviours.

A number of earlier studies investigated a similar demographic of participants using smaller samples although results were not entirely consistent. In agreement with Frazier et al. (2014), some studies have reported that girls have more socio-communication symptoms and lower cognitive and language ability (Carter et al., 2007; Lord et al., 1982). Hartley and Sikora (2009) reported that in a group of toddlers (N > 200), girls were more impaired on the communication domain, but less impaired on the restricted interests/repetitive behaviours domain, than boys. This reduced impairment in females compared to males on the restricted interests/repetitive behaviours domain has been replicated in several previous studies with children and adolescents (Bölte et al., 2011; McLennan et al., 1993; Mandy et al., 2011; Park et al., 2012; Solomon et al., 2012; for a review, see Van Wijngaarden-Cremers et al., 2014) although a recent study with boys and girls under the age of 5 years reported no significant differences in number of symptoms in this domain (Harrop et al., 2015).

Reports of differences in ASD presentation between male and female adults are far more limited. Three studies have included participants ranging in age from childhood through to adulthood. Of these, two reported no significant sex differences in any domain: one included a sample with intellectual disability aged 3–30 years (Pilowsky et al., 1998) and the other included a group without intellectual disability aged 5–20 years (Holtmann et al., 2007). The third, including a sample of ASD individuals aged 6–36 years, found age-related differences: in early development, males had more severe social difficulties than females, but in adolescence and adulthood, females exhibited more severe social and communication difficulties than males (McLennan et al., 1993). These studies were important first steps in investigation of sex differences across the lifespan, but conclusions were inconsistent and may have been limited by small numbers (N < 42/group) and wide age ranges within the samples.

Only one other study has investigated gender and diagnosis in adults: Lai et al. (2011) investigated 62 adults (aged 18–45 years) with previous diagnoses of high-functioning autism or Asperger syndrome. They reported that ASD females had fewer repetitive and stereotyped behaviours than males both in childhood (reported retrospectively) and currently. Females exhibited fewer social-communication symptoms in adulthood although no significant sex differences were detected during childhood.

There are other factors, besides age and level of intelligence, which are an integral part of diagnostic evaluation but have been largely overlooked in previous studies. First, sex differences in symptom profile may vary by diagnostic subtype, yet the majority of studies only include individuals that meet ‘full-ASD’ criteria – that is, they have a diagnosis of Asperger syndrome, childhood autism or high-functioning autism. In the clinical setting, a significant number of people have a ‘partial-ASD’ diagnosis – pervasive developmental disorder–unspecified (PDD-unspecified, or ‘other PDD’) or atypical autism – and these individuals are part of the autistic spectrum as currently defined, and eligible for services and support. One study examined symptom presentation in a sample of high-functioning children referred for an ASD assessment (Mandy et al., 2011) and reported that relatively more males were diagnosed with a full-ASD subtype and relatively more females were diagnosed with a partial-ASD subtype although the difference narrowly missed significance. Beyond relative rates of diagnostic subtypes, a sex-subtype interaction may affect manifestation of autistic traits (Lai et al., 2015). For instance, a recent large-scale study pooling four datasets, each including multiple clinical sites, demonstrated that symptomatic differences between boys and girls on the autistic spectrum vary by dataset (Howe et al., 2015). The authors suggested that the results could have been affected by ascertainment strategies, such that the clinical samples included participants of varying degrees of autistic symptoms. Thus, the validity of extrapolating results from studies of ‘text-book’ cases of childhood autism and Asperger syndrome to the wider autistic spectrum is uncertain in children, and has yet to be examined in adults. Furthermore, ASD subtype diagnoses may provide a useful basis for developing individualized treatment plans, and clarification of potential differences is pertinent because upcoming modifications to the ICD diagnostic system are expected to follow the lead of the DSM-5 by collapsing diagnostic subtypes into one ‘ASD’ diagnosis; therefore, this information may not be available in the future.

Second, co-morbid psychiatric conditions are common in ASD (Hofvander et al., 2009; Joshi et al., 2013; Russell et al., 2005, 2015) and symptoms of ASD are often difficult to disentangle from additional or alternative conditions (Mazzone et al., 2012; Rydén and Bejerot, 2008). This may lead to inaccurate diagnoses (Attwood, 2007) or misguided referrals to specialist clinics. A recent epidemiological study has demonstrated that diagnostic rates of certain common psychiatric conditions – in particular mood and anxiety disorders – are higher in women than men in the general adult population (Kessler et al., 2012), but it is unclear whether sex differences translate to the autistic spectrum, or whether additional mental health conditions influence sex differences in manifestation of autistic symptomology (Lai et al., 2015). It is also of clinical importance to establish what mental health conditions are commonly diagnosed in patients with suspected ASD, but who do not go on to receive a diagnosis of ASD.

To summarize, in this study, we examine whether sex influenced the diagnostic evaluation of ASD in a sample of individuals who were referred to a national specialist clinic for an ASD assessment for the first time in adulthood. We addressed the following four specific aims.

1. To compare the rates of positive ASD diagnoses, and characteristics (age, intelligence, ASD subtype and additional mental health diagnoses), of men and women referred for an ASD assessment.
2. To examine sex differences in type and severity of ASD core-symptoms across the autism spectrum.
3. To examine the moderating effects of diagnostic subtype, the presence of additional psychiatric conditions and IQ, on any sex/core-symptom interactions.
4. To compare characteristics (age, alternative mental health diagnoses) of males and females with suspected ASD, but who did not receive a diagnosis of an ASD.

## Method

### Participants

The initial sample included 1244 individuals aged 18–75 years (inter-quartile range of 22–39 years); 935 males and 309 females. These adults were consecutively assessed for ASD for the first time in a specialist national tertiary ASD clinic between April 2003 and April 2014 (Behavioural Genetics and Adult Autism Clinic, The Maudsley Hospital). People can be referred by their local family physician/general practitioner or consultant psychiatrist for assessment of possible ASD in adulthood and referrals are accepted from both the local community and across the United Kingdom.

Ethical approval was granted by the National Research Ethics Committee, London (12/LO/07990). In an additional 54 cases, diagnosis was inconclusive due to severe psychotic or depressive symptoms, non-compliance or history of major head injury; these individuals were excluded from the study. There were no significant differences in sex distribution between the excluded cases and the full sample.

### Clinical assessment

Assessment included a detailed neuropsychiatric assessment by a multidisciplinary clinical team with expertise in ASD: a consultant psychiatrist, +/− junior doctor and a research-reliable ADI-R/ADOS-G administrator (nurse, psychologist or doctor).

Each patient’s history and clinical information was reviewed on the day of their appointment and they completed a psychiatric clinical interview and ADI-R/ADOS-G assessment (lasting 1–4 h, with breaks as necessary). The ADI-R, lasting 1.5–3 h, is a semi-structured parent/caregiver interview designed to assess and quantify a developmental history of autism-specific behaviours (Lord et al., 1994). The ADI-R was completed if the patient provided consent, and if a parent/early childhood caregiver was available. If it was not possible to complete an ADI-R, or additional information was required to determine diagnosis, an ADOS-G (module 4) was completed. The ADOS-G is a standardized assessment conducted with the patient that lasts 40–60 min. It involves a semi-structured interview interspersed with activities and tasks intended to elicit behaviours associated with ASD. In all, 630 individuals were assessed using the ADI-R, 408 were assessed with the ADOS-G and 206 were assessed using both ADI-R and ADOS-G.

The presence or absence of an ASD diagnosis was made in a diagnostic meeting attended by all members of the clinical team that conducted the assessment, who determined by consensus whether each criterion on the ICD-10R ASD algorithm was fulfilled or not. In line with ICD-10R guidelines, for a patient to meet full ICD-10R criteria for autism, a total of at least six symptoms must be present – either currently or by history – with at least two from the ‘social interaction’ domain and one from each of the ‘communication’ and ‘restricted and repetitive interests’ domains, and symptoms noted before the age of 3 years. They were diagnosed with childhood autism or high-functioning autism (if they exhibited a language delay) or Asperger syndrome (if there was no evidence of a language delay). If a patient differed from the ICD-10R autism criteria either in age of onset (i.e. later than 3 years of age) or number of symptoms (e.g. a lack of sufficient demonstrable abnormalities in one or two of the three ASD domains, despite characteristic abnormalities in other area(s)), they were diagnosed with atypical autism. If a patient’s history and presentation was in keeping with an ASD but there was a lack of adequate information, they were diagnosed with PDD-unspecified.

Of the 1244 referrals, 874 (70%) were diagnosed with an ASD. Of these, 219 (25%) participants were subtyped as childhood autism or high-functioning autism, 429 (49%) as Asperger syndrome, 154 (18%) as atypical autism and 72 (8%) as PDD-unspecified. Except when stated otherwise, for this article, participants with childhood autism, high-functioning autism and Asperger syndrome were subsumed into a ‘full-ASD’ diagnostic subgroup, and those with atypical autism and PDD-unspecified and were subsumed into a ‘partial-ASD’ diagnostic subgroup.

Additional mental health conditions were diagnosed in accordance with the ICD-10R (with the exception of adult attention deficit hyperactivity disorder (ADHD)) which, in keeping with UK guidelines, was assessed using *Diagnostic and Statistical Manual of Mental Disorders* (4th ed., text rev.; DSM-IV-TR).

Neuropsychological testing was completed in 319 participants either for their clinical care if intellectual disability or a significant lacuna in cognitive function was suspected (248 participants completed the Wechsler Adult Intelligence Scale-III (WAIS-III; Wechsler, 1997)) or as part of associated research projects (71 participants completed the Wechsler Abbreviated Scale of Intelligence (WASI; Wechsler, 1999)).

### Data analyses

To address Aim 1, chi-square analyses were employed to compare rates of ASD diagnosis. T-tests were used to compare age and IQ (where available) of ASD participants. In order to focus on symptom profile in high-functioning adults with ASD, participants with confirmed IQ < 70 in any domain (N = 29), and participants where an intellectual disability was suspected but testing could not be completed (N = 17) were excluded from the following analyses, and the final high-functioning ASD sample size included 639 males and 188 females. T-tests were then used to examine sex differences in rates of ASD subtype diagnoses and presence of additional mental health conditions (Table 1).

**Table 1.** Outcome of ASD assessment: age, intelligence, ASD subtype and additional mental health diagnoses in ASD participants; alternative mental health diagnoses in non-ASD participants.

		N	Males	Females	Gender difference	Effect size (Cohen’s d)
Outcome of ASD assessment	% of ASD positive referrals	M: 935, F: 309	71.8%	65.7%	x^2^ = 4.09, *p* = 0.04	0.12
	Mean age of ASD diagnosis	M: 671, F: 203	31.2 years	30.2 years	t = 1.09, *p* = 0.28	0.09
	Suspected or confirmed IQ < 70	M: 671, F: 203	4.8%	7.4%	x^2^ = 2.10, *p* = 0.15	0.05
	Average VIQ	M: 226, F: 56	101.1 (SD: 17.2)	96.3 (SD: 19.3)	t = 1.79, *p* = 0.08	0.26
	Average PIQ	M: 223, F: 56	95.2 (SD: 17.9)	92.0 (SD: 19.1)	t = 1.17, *p* = 0.24	0.17
	Average FIQ	M: 163, F: 41	99.4 (SD: 17.6)	92.4 (SD: 20.2)	t = 2.18, *p* = 0.03	0.37
ASD subtype	Full-ASD diagnoses (as % of all ASD diagnoses)	M: 639, F: 188	75.4%	70.2%	x^2^ = 2.07, *p* = 0.15	0.05
	Partial-ASD diagnoses (as % of all ASD diagnoses)		24.6%	29.8%		
Additional mental health diagnoses in ASD participants	% with any additional mental health diagnosis	M: 639, F: 188	57.6%	61.2%	t = −0.99, *p* = 0.33	0.08
	% with ADHD		12.5%	13.3%	t = −0.28, *p* = 0.78	0.02
	% with social phobia		11.2%	14.3%	t = −1.15, *p* = 0.25	0.10
	% with OCD		17.8%	19.7%	t = −0.47, *p* = 0.64	0.10
	% with any anxiety disorder		40.8%	46.3%	t = −1.46, *p* = 0.15	0.12
	% with any depressive disorder		21.9%	20.2%	t = 0.43, *p* = 0.67	0.04
Alternative mental health diagnoses in non-ASD participants	% with any alternative mental health diagnosis	M: 264, F: 106	54.1%	62.3%	t = −0.66, *p* = 0.51	0.08
	% with ADHD		11.4%	6.6%	t = 1.61, *p* = 0.11	0.19
	% with social phobia		5.7%	17.0%	t = 3.50, *p* = 0.001	0.40
	% with OCD		11.0%	12.3%	t = −0.25, *p* = 0.81	0.03
	% with any anxiety disorder		29.2%	41.5%	t = 2.30, *p* = 0.02	0.27
	% with any depressive disorder		22.7%	23.6%	t = −0.17, *p* = 0.86	0.02

VIQ: verbal IQ; PIQ: performance IQ; FIQ: full-scale IQ; ADHD: attention deficit hyperactivity disorder; OCD: obsessive compulsive disorder.

‘Full-ASD diagnosis’ includes Asperger syndrome, childhood autism and high-functioning autism. ‘Partial-ASD diagnosis’ includes pervasive developmental disorder–unspecified and atypical autism. ‘Any anxiety disorder’ includes phobic disorders, OCD, generalized anxiety disorder, mixed anxiety and depression, social anxiety. ‘Depressive disorders’ include bipolar affective disorder; mild, moderate or severe depressive episode; mixed anxiety and depression; recurrent depressive disorder; dysthymia.

To address Aim 2, sex differences in domain scores of the ADI-R (social, communication, repetitive and restricted interests and behaviours) and ADOS-G (social, communication, stereotyped behaviours and restricted interests) were examined using t-tests. To Bonferroni-correct for multiple comparisons, we considered *p* values of less than 0.006 to be significant (Table 2).

**Table 2.** Core domain scores for high-functioning ASD males and females.

Number of participants	Test and domain	Male	Female	Gender difference	Effect size (Cohen’s d)
ADI-R: N = 320 males, 90 females	ADI-R social	13.2 (6.1)	12.6 (6.3)	t = 1.0, *p* = 0.3	0.10
	ADI-R communication	9.9 (4.7)	9.7 (4.3)	t = 0.4, *p* = 0.7	0.07
	ADI-R repetitive behaviours and restricted interests	3.6 (2.1)	2.9 (2.1)	t = 3.4, *p* = 0.001	0.33
	ADI-R total	26.7 (11.0)	25.1 (10.8)	t = 1.4, *p* = 0.1	0.15
ADOS-G: N = 203 males, 63 females	ADOS-G social	7.9 (2.8)	7.4 (2.8)	t = 1.7, *p* = 0.1	0.21
	ADOS-G communication	3.5 (1.8)	3.0 (1.6)	t = 2.3, *p* = 0.02	0.22
	ADOS-G restricted interests and behaviours	1.6 (1.5)	1.6 (1.4)	t < 0.1, *p* = 0.9	0.06
	ADOS-G social + communication	11.4 (4.1)	10.4 (3.8)	t = 2.2, *p* = 0.03	0.25

ADI-R: Autism Diagnostic Interview–Revised; ADOS-G: Autism Diagnostic Observation Schedule–Generic.

Mean and standard deviation (in brackets) of scores.

**Figure 1.**
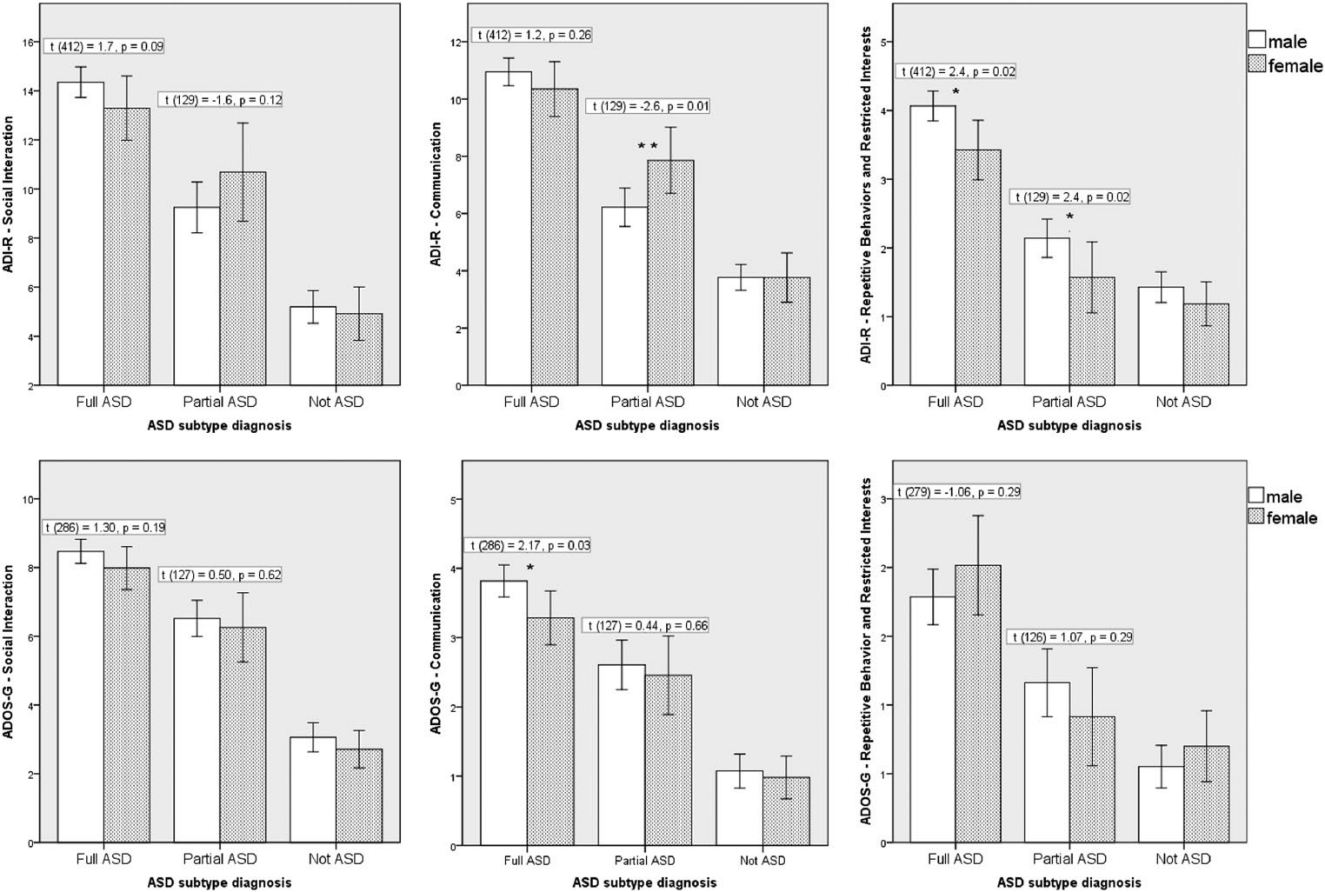
Mean scores on the core domains of the ADI-R, split by sex and diagnostic subtype. Significant interactions were found between sex and diagnostic subtype (full-ASD/partial-ASD) in the communication domain (*p* = 0.02) and in total ADI-R score (*p* = 0.04), and a marginal interaction was found in the social domain (*p* = 0.06).

To address Aim 3, multivariate analyses of variance (MANOVAs) were conducted with sex (male/female) and diagnostic subtype (full-ASD diagnosis/partial-ASD diagnosis) as fixed factors. First, ADI-R domain scores were entered as dependent variables. Post hoc t-tests were performed on significant interactions. The same analyses were conducted with scores from the ADOS-G (Figure 1).

The MANOVAs were repeated including only the ‘full-ASD’ group, contrasting participants with childhood/high-functioning autism (i.e. those with a language delay; N = 429) and Asperger syndrome (i.e. no language delay; N = 154).

The presence of co-morbid conditions (any additional mental health condition; ADHD; phobic disorder; obsessive compulsive disorder (OCD); any anxiety disorder; any depressive disorder) were entered as covariates in the multivariate models reported above (fixed factors: sex, diagnostic subtype; dependent variables: ADI-R scores, ADOS-G scores).

Finally, an exploratory analysis including all ASD participants with verbal IQ (VIQ) and performance IQ (PIQ) data available (N = 279; this included those with an intellectual impairment) was conducted using a MANOVA with sex and subtype as fixed factors, and VIQ and PIQ as dependent variables (full-scale IQ (FIQ) was not included because for many ASD individuals FIQ was not computable due to the discrepancy between VIQ and PIQ).

To address Aim 4, t-tests were used to compare age, ADI-R and ADOS-G scores, and presence of alternative mental health conditions between men and women who were not diagnosed with an ASD (Table 1; Figure 1).

## Results

The outcome of the ASD assessments are presented in Table 1 (age, intelligence, ASD subtype and additional mental health diagnoses in ASD participants; alternative mental health diagnoses in non-ASD participants).

### Aim 1: characteristics of ASD participants

Of the initial 1244 participants, 70% were diagnosed with an ASD (671 males and 203 females; a ratio of 3.3:1). The proportion of participants who received a positive ASD diagnosis was significantly higher in males (72%) compared to females (66%; x^2^ = 4.09, *p* = 0.04, d = 0.12). The mean age was 31.0 years (SD = 11.1) and there was no significant sex difference in the age of ASD diagnosis (N = 874; Table 1).

There was no significant difference between the proportion of males and females who had an IQ below 70 in FIQ, PIQ or VIQ; however, males had significantly higher FIQ than females (*p* = 0.03, d = 0.37) and marginally higher VIQ (*p* = 0.08, d = 0.26).

ASD subtype and additional mental health conditions in high-functioning ASD (N = 827): the ratio of males to females in the full-ASD subtype was 3.7:1, and in the partial-ASD subtype the ratio was 2.8:1. Therefore, relatively more males were diagnosed with full-ASD when compared to those with partial-ASD although the difference was not significant, (*p* = 0.15, d = 0.05). There were no sex differences in proportion of ASD participants who received any additional mental health diagnosis (Table 1; all *p*s > 0.3).

### Aim 2: sex differences in core-symptom profiles in high-functioning ASD (N = 827)

After Bonferroni corrections, the only difference that reached significance was in the repetitive behaviours and restricted interests domain of the ADI-R, with males scoring higher than females, t(526) = 3.27, *p* = 0.001, d = 0.33. All other comparisons were non-significant (*p*s > 0.02).

### Aim 3: interactions between sex, diagnostic subtype and core-symptoms

As expected, the MANOVA confirmed that on average the full-ASD participants scored significantly higher than partial-ASD participants in all ADI-R domains (all *p*s < 0.001, all $\eta_{p}^{2}>0.05$). The effect of sex was only significant for the repetitive behaviours and restricted interests domain (male > female; F(1) = 7.62, *p* = 0.006, $\eta_{p}^{2}=0.01$). There was a significant interaction between sex and diagnostic subtype in ADI-R communication domain (F(1) = 5.28, *p* = 0.02, $\eta_{p}^{2}=0.01$), and a marginal interaction in the ADI-R social domain (F(1) = 3.52, *p* = 0.06; $\eta_{p}^{2}=0.01$; Figure 1). The interaction in the repetitive behaviours domain was non-significant (*p* = 0.9). Post hoc t-tests (Figure 1) confirmed that in the full-ASD group the average male score was higher than the average female score on the social and communication domains. Conversely, in the partial-ASD group, the average female score was higher than the average male on the social and communication domains. In the repetitive behaviours and restricted interests domain, the average male score was significantly higher than the average female score in all ASD subtypes.

In the ADOS-G, the MANOVA confirmed the expected effect of diagnostic subtype (*p* < 0.001, $\eta_{p}^{2}=0.1$) but no significant effect of sex (*p* = 0.5). The interaction between sex and diagnostic subtype was not significant (*p* = 0.14).

#### Asperger syndrome versus childhood/high-functioning autism

In the ADI-R, there were significant effects of subtype in the communication domain (childhood/high-functioning autism > Asperger; F(1) = 23.2, *p* < 0.001, $\eta_{p}^{2}=0.05$) and in the social interaction domain (childhood/high-functioning autism > Asperger; F(1) = 17.5, *p* < 0.001, $\eta_{p}^{2}=0.04$). The effect of sex was only significant for the repetitive behaviours/restricted interests domain (male > female; F(1) = 9.18, *p* = 0.003, $\eta_{p}^{2}=0.02$). There were no significant interactions between subtype and sex. In the ADOS-G, there was a significant effect of subtype only in the repetitive behaviours/restricted interests domain (Asperger > childhood/high-functioning autism; F(1) = 6.26, *p* = 0.01, $\eta_{p}^{2}=0.02$). Significant effects of sex were evident in the communication domain (male > female; F(1) = 4.14, *p* = 0.04, $\eta_{p}^{2}=0.02$), but again, there were no significant interactions between subtype and sex.

#### Additional mental health conditions

The significance of results of the multivariate models was unchanged when additional mental health conditions were added as covariates.

#### IQ

This analysis was conducted with all ASD participants where VIQ and PIQ data were available (N = 279), including those with an intellectual impairment who were excluded from previous analyses. There was no significant effect of sex (male IQ > female IQ; F(2) = 2.47, *p* = 0.09, $\eta_{p}^{2}=0.02$), no significant effect of subtype (*p* > 0.3), and no significant interaction between sex and subtype (*p* > 0.4).

### Aim 4: non-ASD participants (N = 370)

The non-ASD participants were significantly older than the ASD participants (t(1242) = 4.70, *p* < 0.001, d = 0.3) (mean age = 34.3 years, SD = 12.0 years).

There were no significant differences on any domains of the ADI-R or ADOS-G between males and females who were not diagnosed with ASD (N = 370, all *p*s > 0.3; Figure 1).

In all, 62% of non-ASD women and 54% of non-ASD men were diagnosed with at least one alternative mental health condition. Significantly, more females than males were diagnosed with social phobia, t = 3.5, p = 0.001 (d = 0.4). Females were also diagnosed with anxiety disorders more frequently than males, t = 2.3, *p* = 0.02 (d = 0.3) although correcting for multiple comparisons renders this result non-significant.

## Discussion

In this study, we examined sex differences in a clinical sample of adults referred for an assessment of ASD to determine whether sex influenced diagnostic evaluation. Participants had been referred for an ASD assessment for the first time in adulthood and the majority had no intellectual disability. In answer to our first aim, more men (72%) than women (66%) received a positive ASD diagnosis of any subtype; this difference was significant although the effect size was small. If we accept that the higher rate of ‘incorrect’ referrals in women exists, it could either be due to general health practitioners/psychiatrists being less clear about how ASD manifests in adult females, or it could be due to an under-diagnosis of women and thus a need to adjust the diagnostic criteria. The sex ratio in the high-functioning ASD group was 3.4 men to 1 woman. There were no sex differences in age or presence of additional mental health conditions (58% of men, 61% of women had at least one co-morbid diagnosis), but males had marginally higher IQ scores and there was a trend towards a higher proportion of males in the full-ASD subtype.

In response to our second aim, there were notable differences in core-symptom profiles; overall, both sexes exhibited a similar degree of socio-communicative symptoms but men exhibited more restricted behaviours and repetitive interests than women. However, in response to our third aim, sex differences in core-symptomology varied by subtype.

### Sex differences in core-symptom profiles: evidence for differing manifestations of the ASD phenotype

The finding of no significant sex differences in socio-communicative symptoms in the ASD group as a whole is in line with a review by Van Wijngaarden-Cremers et al. (2013), but contrasts with studies reporting that females have more (Carter et al., 2007; Frazier et al., 2014; Hartley and Sikora, 2009; Lord et al., 1982; McLennan et al., 1993) or less (Lai et al., 2011) symptoms than males. This discrepancy is potentially reconcilable by the fact that the studies listed include different age groups and different criteria for inclusion since our results indicate that socio-communicative symptoms are not constant across the spectrum: in the full-ASD group, adult males exhibited more socio-communicative symptoms than females, but in the partial-ASD group the reverse was true. By contrast, in all diagnostic subtypes, males scored significantly higher than females on the repetitive behaviours/restricted interests domain of the ADI-R. This is widely consistent with previous research (Bolte et al., 2011; McLennan et al., 1993; Mandy et al., 2011; Park et al., 2012; Solomon et al., 2012; Van Wijngaarden-Cremers et al., 2013) although contrasts with recent evidence from young children (Howe et al., 2015), and suggests an alternative explanation for the results from the socio-communicative domains: females frequently have prominent symptoms in the socio-communicative domains but reduced symptoms in the repetitive behaviours/restricted interests domain. This places them into the ‘partial-ASD’ diagnostic category and means that males and females with the same diagnostic label often have very different symptom profiles. Of course, ASD is a highly heterogeneous condition so variability within subtypes is to be expected; however, these results contribute to emerging evidence for sex-specific manifestations of the autism phenotype. Specifically, ASD females without an intellectual disability typically exhibit fewer repetitive behaviours and restricted interests than their male counterparts with comparable socio-communicative impairment.

### Sex differences in core-symptom profiles: implications for efficacy of diagnostic tools

Our approach cannot rule out the possibility that women do not exhibit ‘fewer’, but that they exhibit ‘different’, repetitive behaviours or restricted interests. This is because current assessment tools, such as the ADI-R and ADOS-G, have been designed to measure the symptoms that define ASD, therefore only serve to confirm or reject the presence of what we describe as ‘ASD traits’. If females (or males) actually manifest symptoms not currently included in the algorithm, no current assessment tool or diagnostic algorithm will detect that. This problem is referred to as the ‘nosological (how autism is defined) and diagnostic (how autism is identified) challenge’ of ASD research (Lai et al., 2015).

Use of qualitative methods to investigate sex-typical traits could contribute useful information to this debate. However, to date, few studies have documented how repetitive behaviours and restricted interests actually differ between males and females. One possibility is that girls are more likely to have socially accepted special interests that may mask the atypical nature of the interest (Kopp and Gillberg, 1992; Lai et al., 2015). For example, a parent may report that their daughter liked playing with dolls, but when probed about how they ‘played’ it could become apparent that every session involved brushing the hair again and again, with little flexibility or imagination. Moreover, we propose that circumscribed interests in males could actually be over-identified due to preconceptions about common interests in ASD boys. For example, a parent may report their son was very keen on trains or dinosaurs, this could be over-interpreted as a ‘special interest’, but on further questioning it may emerge that in this particular individual the trains/dinosaurs interest was little more than an age-appropriate phase that did not interfere with other interests. Thus, clinicians should be careful of stereotyping observed behaviours. Identifying common examples of restricted interests and repetitive behaviours in both sexes across the spectrum in both childhood and adulthood may alleviate this problem.

Additional future investigations could focus on developmental differences between males and females on the spectrum across the lifespan (Lai et al., 2015). In our report, more prominent sex differences were identified by the ADI-R (focusing mainly on childhood symptoms) than the ADOS-G (which focuses on current symptoms). While we note that the ADOS-G is not consistently sensitive to repetitive and restricted behaviours in male or female adults (hence, scores in this domain are not required for diagnosis), the results suggest further investigation into change in symptom presentation over the lifespan warrants research. Longitudinal methods would be ideal to eliminate the effects of parental bias, whereby parental tolerance and recall of perceived difficulties in early childhood may vary across gender which would influence results of the ADI-R but not the ADOS-G. However, behavioural adaptations and learned skills that may contribute to lower present-state ADOS-G scores in some adults with ASD should be considered. Ultimately, we should aim to improve guidelines for general healthcare professionals, parents and teachers, and introduce clear examples for both sexes into diagnostic algorithms.

This issue is also relevant to the manifestation and development of socio-communicative symptoms in males and females. Our result of ‘no overall differences in number of socio-communicative symptoms’ was likely masking differences in more fine-grained socio-communicative symptoms – hence the evident contrasts between subtypes. A recent paper demonstrated that boys and girls on the spectrum that were matched for overall level of core-symptomology contrasted in terms of what factors were associated with play skills (Harrop et al., 2014). The authors reported that in boys, the social-communication skill of ‘initiating behavioural requests’ was associated with non-verbal IQ and language ability, but in girls, it was ‘responding to behavioural requests’ that was associated with non-verbal IQ. The authors note that the contrasting correlations did not survive Bonferroni corrections, but nevertheless they promote investigations into detailed components of core-symptoms with attention to potential sex differences in how these may relate to other cognitive functions.

In this study, we investigated how autistic traits related to additional cognitive and behavioural symptoms in two respects: presence of additional mental health conditions and intelligence. Regarding IQ, our results were largely in line with previous research reporting that females with a diagnosis of ASD tend to have a lower IQ than males (Fombonne, 2005), and non-significant interactions between IQ and ASD subtype provided no evidence for variation across the spectrum. Regarding additional mental health diagnoses, our results indicated no sex differences in prevalence of additional psychopathologies in the ASD group, or interactions with core-symptomology, at the time of assessment. Nevertheless, we note that it is still possible that sex differences exist regarding historical diagnoses, rates of previous misdiagnosis and patterns of evolving diagnosis across the lifespan. All these have potential implications for diagnostic practice.

We also contrasted core-symptom presentation between Asperger syndrome (full-ASD with no language delay) and childhood autism/high-functioning autism (full-ASD with a language delay). Group differences were evident: Asperger syndrome participants exhibited significantly more social interaction symptoms, but fewer communication symptoms than their childhood autism/high-functioning autism counterparts. This warrants further investigation and has implications for collapsing the two diagnostic subtypes, and the two domain categories, in the DSM-5 (Wilson et al., 2013) and forthcoming ICD-11. However, no sex-subtype interactions emerged; thus, we found no evidence that a language delay differentially affects the development of core-symptoms in late-diagnosed males and females.

In general, our results raise the issue of ‘spread’ of symptoms versus ‘severity’ of symptoms when using diagnostic algorithms. Currently, an individual with moderate symptoms spread across all domains will qualify for a diagnosis (perhaps a typical male profile), but those with severe symptoms focused in one domain may not (perhaps a typical female profile). Some of these people may qualify for an alternative, possibly more appropriate, diagnosis but others may miss out on a diagnosis altogether and hence not receive any services or support. In the DSM-5 (2014), social and communication symptoms are collapsed to a single domain, and an individual must fulfil three out of three criteria (and two out of four criteria in the repetitive/restricted behaviour domain) to qualify for the ASD diagnosis. Thus, there is a strict cut-off for minimum ‘spread’ of symptoms. The impact of the new system is yet to be established, but a study analysing clinic outcomes retrospectively for 150 adults suggested that 44% of participants that met criteria for any ASD using the ICD-10R, and 22% that met DSM-IV-TR criteria for Asperger syndrome/autistic disorder, would not qualify for a diagnosis of ASD under the DSM-5 (Wilson et al., 2013). The same study reported no differences in rates of men and women who would qualify for diagnoses under current and new systems although this warrants replication with prospective data. Regarding varying levels of symptom severity within the diagnostic category of ASD, the DSM-5 has introduced three ‘severity levels’ to be allocated on the basis of accompanying intellectual impairment, language impairment or known medical/genetic/environmental factors. However, this does not deal with differences in specific symptom severity and core-symptom profiles, which may be a factor for consideration in future diagnostic tools. In addition, we note that the DSM-5 (and likely the forthcoming ICD-11) relies heavily on retrospective data when examining adults. Following the earlier proposal that parental recall may differ for girls and boys, females may again be at a disadvantage in terms of fulfilling criterion and being adequately diagnosed.

### Implications for service design

This report has implications for ASD services that continue to evolve in the wake of the Autism Act (Her Majesty’s Government, 2009) and National Institute for Clinical Excellence guidelines (NICE, 2012; Wilson et al., 2014). ASD is currently the only mental health disorder with dedicated legislation in the United Kingdom, but the resulting increase in demand for ASD services coincides with a reduction in available resources in the healthcare system. Since specialized assessments are time-consuming and costly to both patients and service providers (Murphy et al., 2011), it is useful to know what could underlie ‘errors’ – that is, referrals that do not result in an ASD diagnosis. In response to our fourth aim to compare characteristics of those patients who were not diagnosed with ASD, 54% of men and 62% of women were diagnosed with an alternative condition; this discrepancy was driven by a significantly higher rate of social phobia in women. There is clear overlap between symptoms of social anxiety and ASD; for example, behaviours common to both include social withdrawal and being quiet in social situations (Eriksson et al., 2013). However, important distinctions can be made, for example, adults with social phobia may be anxious that they are socially inept, but actually these skills and knowledge are not lacking. By contrast, adults with ASD may lack knowledge about how to act appropriately in social situations, and they may or may not have insight into this deficit (Bejerot et al., 2014). Continued investigation into how symptoms of social phobia and ASD differ, in particular in females, along with more sensitive and readily available screening tools, could help general practitioners and psychiatrists avoid errant referrals and appropriately identify diagnoses and access to management options.

Nevertheless, we stress that in this sample around 70% of patient referrals were accurate – that is, assessment confirmed the suspected ASD diagnosis – this is a substantial improvement on the 50% accuracy rate (Murphy et al., 2011) and 56% accuracy rate (Russell et al., 2015) that were reported from the same national clinic with data from 4 years ago and 3 years ago, respectively. Thus, awareness and detection of ASD symptomology by general health professionals does seem to be improving.

### Strengths, limitations and future research

Concerning limitations, this sample included only people not diagnosed during childhood and therefore the sample may be skewed towards people whose childhood symptoms were subtle, overcome by compensatory factors or undetected for other reasons. The extent to which data reported here can be generalized to the ‘early-diagnosed’ ASD population remains unclear. In particular, whether sex differences differ by early versus late-diagnosed individuals remains unknown and warrants further investigation.

Formal IQ testing was not completed for the majority of participants, but instead intelligence levels were assumed to be in the normal range (IQ > 70) unless the clinicians – who were highly experienced in working with adults with intellectual disabilities – had any reason to suspect otherwise. The relationship between ASD symptom profile and IQ level could therefore not be investigated within this high-functioning sample. Analysis of non-core-symptoms was beyond the scope of this report; however, previous research has indicated differences between males and females in several specific domains including executive functioning (e.g. Bölte et al., 2011; Lai et al., 2012; Lemon et al., 2011), perceptual attention to detail and motor function (Lai et al., 2012), adaptive skills (Frazier et al., 2014), autobiographical memory (Goddard et al., 2014) and sleep habits (Hartley et al., 2009). Such factors could interact with core-symptom presentation and may contribute to a definition of sex-specific manifestations of the ASD phenotypes.

Finally, we acknowledge that despite the expertise of the clinical team and the use of adequate instruments, diagnostic misses of ASD and/or a failure to detect certain symptoms was possible. Not all alternative disorders could be assessed since appointments were completed in a single day. Attachment disorder/personality disorder, for example, requires further investigation and was likely to be an accurate diagnosis for some of the non-ASD participants. The possibility that differing rates of attachment disorder in men and women who are referred for an ASD assessment remains open.

Concerning strengths, this study included a relatively large sample size in comparison to the existing literature, and every participant underwent an assessment with specialist clinicians using best available diagnostic tools. The sample is from a national tertiary clinic and is likely to be representative of the adult population presenting with autism-like mental health problems in the United Kingdom. The inclusion of adults with diagnoses across the autistic spectrum provides confidence that results are applicable to real clinical settings of adult-diagnostic clinics – an advance on most previous studies that have included only ‘full-ASD’ subtypes. Moreover, the inclusion of participants who were referred for an ASD assessment but were *not* on the autistic spectrum allowed us to explore under what circumstances ASD may be incorrectly identified in males and females.

## Conclusion

We report sex differences in symptom profile in late-diagnosed individuals with ASD, and suggest that men and women may present with different manifestations of the ASD phenotype. Sex appears to influence the diagnostic evaluation of adults, and further research should investigate how this impacts on clinical care, in particular whether males and females respond differently to treatment.
